# Association between Maternal and Foetal Erythrocyte Fatty Acid Profiles and Birth Weight

**DOI:** 10.3390/nu10040402

**Published:** 2018-03-23

**Authors:** Giulia Cinelli, Marta Fabrizi, Lucilla Ravà, Fabrizio Signore, Pamela Vernocchi, Michela Semeraro, Cristina Vallone, Rosalba Lanciotti, Marta Ciofi degli Atti, Melania Manco

**Affiliations:** 1 Research Unit for Multifactorial Diseases and Complex Phenotypes, Bambino Gesù Children Hospital, IRCCS (Istituto di Ricovero e Cura a Carattere Scientifico), viale di San Paolo 15, 00146 Rome, Italy; giuliacinelli@nutrizionista.email (G.C.); marta.fabrizi@gmail.com (M.F.); michela.semeraro@opbg.net (M.S.); 2 Clinical Epidemiology, Bambino Gesù Children’s Hospital, IRCCS (Istituto di Ricovero e Cura a Carattere Scientifico), P.zza S. Onofrio 4, 00165 Rome, Italy; lucilla.rava@opbg.net (L.R.); marta.ciofidegliatti@opbg.net (M.C.d.A.); 3 Department of Obstetrics and Gynaecology, Misericordia Hospital Grosseto, Usl Toscana Sud-est, 58036 Grosseto, Italy; fabrizio.signore@uslsudest.toscana.it (F.S.); cristina.vallone@uslsudest.toscana.it (C.V.); 4 Unit of Human Microbiome, Genetic and Rare Diseases Area, Bambino Gesù Children Hospital, IRCCS (Istituto di Ricovero e Cura a Carattere Scientifico), viale di San Paolo 15, 00146 Rome, Italy; pamela.vernocchi@opbg.net; 5 Centro Interdipartimentale di Ricerca Industriale Agroalimentare, Università degli Studi di Bologna Cesena, 47521 Cesena, Italy; rosalba.lanciotti@unibo.it; 6 Dipartimento di Scienze e Tecnologie Agro-alimentari, Università degli Studi di Bologna, Piazza Goidanich 60, 47521 Cesena, Italy

**Keywords:** birth weight, small for gestational age, fatty acids, saturated fatty acids

## Abstract

Regular foetal development is crucial for assuring good health status in the offspring. The quality and quantity of maternal dietary fatty acids (FAs) can affect growth. The study aimed to: (1) investigate the association of maternal/foetal lipid profiles with birth weight (BW); and (2) compare these profiles in small, appropriate, and large for gestational age (SGA, AGA, and LGA) infants. FAs were measured in erythrocyte membranes using gas chromatography analysis in 607 mother–infant pairs (316 males, 52.1%). In the quantile regression, a significant association between BW and levels of maternal linoleic acid (LA; C18:2, n-6; coefficient: 18.66; *p* = 0.010), arachidonic acid (AA; C20:4, n-6; coefficient: 11.35; *p* = 0.007), docosahexaenoic acid (DHA; C22:6, n-3; coefficient: 29.73; *p* = 0.007), polyunsaturated FAs (coefficient: 8.55; *p* = 0.001), foetal DHA (coefficient: −22.82; *p* = 0.037), and saturated FAs (coefficient: −65.41; *p* = 0.002) was found. Myristic (C14:0) and pentadecanoic acids (C15:0), both maternal (*p* = 0.000; *p* = 0.017) and foetal (*p* = 0.009; *p* = 0.002), and maternal erucic acid (C22:1, n-9; *p* = 0.026) were found at higher levels in SGA infants as compared to AGA ones. Conversely, maternal LA, AA, and omega 6 FAs levels were higher in AGA infants (*p* = 0.037; *p* = 0.003; *p* = 0.026, respectively). Maternal and foetal polyunsaturated and omega 6 FAs levels are positively related to BW, while a lipid profile rich in saturated FAs and erucic acid may influence the risk of SGA.

## 1. Introduction

Regular foetal development during pregnancy is pivotal for assuring good short and long-term health status of the offspring. Both higher and lower intrauterine growth are not only related to problems in the immediate neonatal period, but are also associated with adverse outcomes later in life and likely in future generations [1]. In particular, limited supply of nutrients to foetuses leads them to adapt and change their metabolism and structure in a permanent way. In doing so, both small and large for gestational age new-borns are at increased risk of later morbidity and mortality, e.g., from metabolic disorders like diabetes, hypertension, and cardiovascular disease [2,3,4].

Maternal nutrition is essential for adequate foetal development. The amount and quality of fatty acids (FAs) consumed by the mother during pregnancy can affect foetal programming [5]. Specifically, foetal exposure to trans FAs appears to promote early deleterious effects in the offspring’s health, thereby increasing the individual risk for developing metabolic diseases throughout life [6]. Similarly, the maternal intake of saturated fatty acids (SFAs) seems to trigger alterations in the liver and adipose tissue function associated with insulin resistance and diabetes [6].

A number of reports have examined maternal and cord blood fatty acids composition throughout pregnancy and its association with birth outcomes. Nevertheless, the majority investigated mainly plasmatic concentrations and focused on trans fatty acids or long chain polyunsaturated fatty acids (LC-PUFAs), with scarce attention on SFAs and monounsaturated fatty acids (MUFAs) [7,8,9,10,11,12].

A recent investigation carried on by Meher et al. examined the impact of fatty acids on birth outcomes considering low and normal birth weight (LBW and NBW) infants [13]. To our knowledge, only one study has compared maternal and foetal FAs composition in small, appropriate and large for gestational age (SGA, AGA, and LGA) new-borns. They focused their research on SFAs but using blood samples of a limited cohort [14].

The main aims of this population study were: (1) to evaluate the association between both maternal and foetal erythrocyte lipid profiles and birth weight by considering the full spectrum of fatty acids; (2) to compare maternal and foetal FAs profiles in SGA, AGA, and LGA infants.

## 2. Materials and Methods

### 2.1. Subjects and Study Design

The “Feeding Low-Grade Inflammation and Insulin Resistance of the Foetus” project is a population study of 1000 mother–infant pairs, with the primary aim of evaluating the association at birth between maternal erythrocyte concentrations of FAs and the insulin resistance and low-grade inflammation of the offspring. Details of the study are given elsewhere [15]. In total, 847 mothers completed the study.

Eligibility for mother–infant pairs was defined by gestational age between the completed 37th and 42nd week, Apgar score higher than 7 at five minutes, and absence of genetic disorders.

### 2.2. Anthropometrics and Clinical Evaluation

Descriptions of maternal anthropometric data and clinical evaluations were provided in a previous paper [15]. Maternal pre-pregnancy anthropometry was self-reported. The body mass index (BMI) was calculated in kg/m^2^ and classified according to the World Health Organization (WHO) [16]. Gestational weight gain (GWG) was calculated by subtracting the pre-pregnancy weight from the weight reached at time of delivery, and was classified according to the Institute of Medicine (IOM) guidelines as adequate, inadequate, or excessive [17].

New-borns’ anthropometrics (body weight, BW; birth crown–heel length, BL; and head circumference, HC) were evaluated at birth. BW was measured within 1 h of delivery with an electronic weighing scale and recorded to the nearest 5 g. BL and HC were measured within 1 day of delivery with a Harpenden neonatometer and an inelastic tape, respectively, and recorded to the nearest millimetre [18]. New-borns were defined as being SGA when their birth weights were below the 10th percentile for gestational age and as LGA when their birth weights were above the 90th percentile for gestational age. AGA new-borns were defined as those with birth weights at or above the 10th percentile and at or below the 90th percentile for gestational age [19]. Standard deviation scores (SDSs) for infant weight and height were calculated using the WHO charts (Anthro software program for Windows, version 3.2.2, World Health Organization, 1211 Geneva 27, Switzerland).

The following sociodemographic and anthropometric data for both the parents were collected to estimate socioeconomic status (SES): ethnicity, level of education, employment, smoking, and parity.

### 2.3. Sample Collection

Maternal blood samples were withdrawn under fasting conditions, 12–24 h before giving birth, during pre-partum foetal monitoring. Cord-blood samples (2.5 mL) were collected at birth by venepuncture from the placental portion of the umbilical cord immediately after clamping.

Erythrocyte fatty acids were measured by gas chromatography. A detailed description of the methods for blood collection and FAs analysis has been provided elsewhere [15]. Briefly, the chromatograms were integrated and identified by comparing the retention times and the peak areas with those of a commercial lipid standard and a conjugated linoleic acids mixture. Finally, quantitative data were obtained by interpolation of the relative areas vs. internal standard (methyl-C11) area.

### 2.4. Statistical Analysis

Data are represented as number and percentage in parentheses (%) for categorical variables, or median and interquartile range (IRQ) for continuous variables. The Skewness–Kurtosis test was performed in order to evaluate variable distribution. All the variables had skewed distribution.

Univariable quantile regression analyses were conducted to investigate the association between infant birth weight (BW, the dependent variable) and maternal/foetal erythrocyte lipid profile and infant and parent characteristics (GWG, pre-pregnancy maternal BMI, paternal BMI, offspring sex, maternal age, maternal education level, gestational age, smoking during pregnancy, and parity).

The association between BW and maternal/foetal erythrocyte lipid profile was further explored by developing a multivariable quantile regression model for each FAs while adjusting for infant and parent characteristics, considering only FAs and covariates statistically significantly associated to BW (*p* < 0.2 at univariable analysis). The final multivariable models were determined through a backward approach.

Kruskal-Wallis analysis, with Sidák’s post hoc test, was performed in order to compare the fatty acid profiles in the SGA, AGA, and LGA groups. The Wilkoxon signed-rank test was performed and the Spearman correlation coefficient was calculated to evaluate differences and correlations between maternal and foetal fatty acid compositions in the three different groups (SGA, AGA, and LGA). Results were significant for *p*-value < 0.05.

Statistical analysis was performed through Stata 13.1 software (StataCorp, 4905 Lakeway Drive, College Station, TX, USA).

### 2.5. Ethics Approval

The “Feeding” study was approved by the Ethical Committees of the “*Ospedale Pediatrico Bambino Gesù*” (OPBG) and the *San Camillo Forlanini* Hospital (SCH), in full agreement with the national and international regulations and the Declaration of Helsinki (2000). All the participants signed an informed consent form.

## 3. Results

### 3.1. Subjects

From the initial cohort of 1000 pregnant women enrolled, 153 (15.3%) mothers withdrew from the study (6 genetic diagnoses, 24 childbirth complications not allowing blood collection, 32 personal reasons, 8 miscarriages, and 83 deliveries in different hospitals). No differences were found in age, anthropometrics, and SES of women who participated or withdrew from the study (data not reported).

A complete data set of FAs profiles was available for 694 mother–infant pairs out of 847 (81.9%). For the current analysis, we excluded 41 (5.9%) women with history of diabetes (including gestational diabetes) and 46 (6.9%) mother–infant pairs because of missing data.

A final sample of 607 mother–infant pairs (316 males, 52.1%) was available for the analysis. Most of the women were of normal weight before pregnancy (427, 70.3%) while GWG was inadequate, adequate, or excessive in 27.4%, 40.0%, and 32.6% of women, respectively. All the infants were born at term and 483 (80.5%) of them were classified as AGA, while 49 (8.2%) and 686 (11.3%) were SGA and LGA, respectively. Table 1 shows maternal and foetal characteristics of the sample.

### 3.2. Association between Maternal or Foetal Lipid Profile and Birth Outcomes

Forty FAs were detected using gas chromatography analysis, with lengths ranging from 12 to 22 carbons, and 38 were identified and reported in Table S1. Fatty acids C18:1 n-5, n-4 and C22:3 n-3/C22:4 n-5 were not considered for the present analysis. Table 2 shows the results of the multivariable quantile regression for BW. In terms of foetal long chain saturated fatty acids, higher percentages of myristic (C14:0) and pentadecanoic acids (C17:0) were found to be associated with lower BW. Conversely, higher percentages of maternal LC-PUFAs both omega-3 and omega-6 were associated with higher infant BW at delivery. An inverse association was found between foetal docosahexaenoic acid (DHA, C22:6, n-3) and infant BW.

### 3.3. Maternal and Foetal Lipid Profile in Small for Gestational Age, Adequate for Gestational Age and Large for Gestational Age Infants

Table 3 and Table 4 show maternal and foetal lipid profiles in SGA, AGA, and LGA infants, as percentages. Saturated FAs levels, both maternal and foetal, resulted higher in the SGA group when compared to the AGA group in the post hoc analysis (maternal myristic acid, C14:0: *p* = 0.000; maternal pentadecanoic acid, C15:0: *p* = 0.007; maternal total SFAs: *p* = 0.040; foetal myristic acid, C14:0: *p* = 0.006; foetal pentadecanoic acid, C15:0, *p* = 0.004; foetal eptadecanoic acid, C17:0, *p* = 0.004). Moreover, levels of maternal erucic acid (22:1, n-9) were found to be higher in SGA infants (*p* = 0.003). Trans elaidic acid levels (trans C18:1, n-9) were found to be higher in SGA infants as compared to AGA infants (*p* = 0.046). In contrast, higher percentages of maternal and foetal LC-PUFAs, both omega 3 and omega 6, were detected in AGA new-borns (maternal linoleic acid, LA, C18:2, n-6, *p* = 0.030; maternal eicosapentaenoic acid, EPA, C20:5, n-3: *p* = 0.001; maternal total n-6: *p* = 0.025; foetal eicosadienoic acid, C20:2, n-6: *p* = 0.005; foetal EPA, C20:5 n-3: *p* = 0.039; foetal docosapentaenoic acid, DPA, 22:5 n-3: *p* = 0.032).

Differences between maternal and foetal fatty acid compositions in the three different groups (SGA, AGA and LGA) showed maternal pentadecanoic acid, eptadecanoic (C17:0), trans 18:1, n-7 and PUFAs levels to be higher in the AGA (*p* = 0.035; *p* = 0.008; *p* = 0.000; *p* = 0.000) and LGA (*p* = 0.002; *p* = 0.002; *p* = 0.005; *p* = 0.000) groups but not in the SGA group (*p* = 0.524, *p* = 0.342; *p* = 0.502; *p* = 0.517). Exclusively in the SGA group, erucic acid levels resulted higher in the maternal lipid profile when compared to the foetal group (*p* = 0.032), while DHA levels were higher in the cord blood erythrocytes (*p* = 0.033).

No differences was found in the correlation between maternal and foetal fatty acids in the three group, except for erucic acid (*r* = 0.06, *p* = 0.702) and total SFAs (*r* = 0.06, *p* = 0.665), for which no correlation was found in the SGA group; and trans 16:1 n-7 for which a correlation was found only in the AGA group (*r* = 0.26, *p* = 0.000). 

## 4. Discussion

Our results indicate the following: (1) maternal LC-PUFAs levels are positively associated with birth weight; (2) foetal SFAs are negatively associated with birth weight; (3) both maternal and foetal SFAs levels are higher in the SGA group as compared to the AGA and LGA groups; and (4) maternal erucic acid levels are higher in the SGA group as compared to AGA and LGA groups. Therefore, a maternal lipid profile rich in saturated fatty acid and erucic acid seems to be able to predict giving birth to a SGA infant.

It is difficult to compare the results of the present investigation with previous ones. Indeed, three studies investigated plasma levels of FAs [7,8,11,14]. The four studies performed on erythrocyte membranes [9,10,12,13] basically found overlapping levels of FAs, even though two of them [10,13], compared NBW and LBW infants instead of the SGA and AGA classes, one study focused on few FAs [9], and the remaining study provided comparable data only for cord blood [13].

We found maternal LA, AA, DHA, and total LC-PUFA levels to be positively associated with offspring weight at delivery. Both maternal and foetal LC-PUFA levels were found to be higher in the AGA group when compared to the SGA group. These findings confirm what was previously found on LC-PUFAs with respect to their indispensable role in foetus and infant growth and development [9,10,12,13]. Conversely, DHA was inversely associated with birth weight and, exclusively in the SGA group, levels were found to be higher in infants when compared to mothers. We hypothesise a preferential transfer of this polyunsaturated fatty acid through the placenta due to the increased needs of the foetus. The possible mechanism could be through an up-regulation of mRNA expression in placental fatty acid transporters as a compensatory mechanism in SGA foetuses [20].

A previous study by Bobinski et al., compared the fatty acid profiles in maternal and cord blood in AGA (*n* = 54) and SGA (*n* = 239) infants born at term. No difference was found in maternal lipid profile. Conversely, they found foetal lauric acid (12:0) levels to be higher in SGA infants. They hypothesized the increase level of lauric acid to be a response to increased energy requirements of infants belonging to the SGA group [14]. Recently, Meher et al. investigated the association between maternal fatty acid profile across gestation and cord blood lipid profiles in 46 LBW and 52 NBW infants. They found higher levels of maternal erythrocyte SFAs in women delivering LBW babies and attributed this to the inadequate transfer of these fatty acids through the placenta, contributing to inadequate foetal growth [13]. Our results suggest a negative association between both maternal and foetal SFAs and birth weight. Maternal myristic (C14:0) and pentadecanoic (C15:0) acids, and foetal C14:0, C15:0, and eptadecanoic acid (C17:0) were all detected to be higher in SGA infants when compared to AGA ones. When we examined the SGA group alone we found no statistical difference between foetal and maternal SFAs, in contrast to the AGA and LGA group where maternal SFA levels appeared to be higher. The correlation was positive. Hence, we do not assume decreased placental transfer of these FAs during pregnancy, or their role as energy supply, but, conversely, we hypothesise their over-representation in mothers to reflect the higher level in the foetus and their possible adverse effects on development [21].

In respect to trans fatty acids, we found trans elaidic acid (trans 18:1 n-9) levels to be higher in SGA infants when compared to the AGA ones. Trans FAs are described to be inversely associated to LC-PUFAs in pregnant women and their new-borns and may interfere with metabolism and trans-placental transfer [22]. Previous studies on plasma concentration suggested possible important effects of trans FAs on foetal growth [7].

Finally, even if no association was found between erucic acid and birth weight, we found maternal erucic acid levels to be higher in the SGA group when compared to the AGA and LGA ones. No difference was found for foetal erucic acid. When we examined the SGA group alone, erucic acid levels resulted higher in the mother, and no correlation between the mother and foetus was found.

While erythrocyte FAs may better reflect FAs intake than dietary recalls [23], in mothers they do not necessary reflect diet since SFAs can be synthesized, while PUFAs can be elongated. These mechanisms may be variable between mothers for numerous reasons, including genetic variations. In any case, our previous work showed a strong correlation between maternal and foetal lipid profile, so we assumed maternal erythrocytes FAs to reflect infants ones, and both to be a possible factor influencing infant size at birth [15].

The mechanisms linking maternal and foetal overexpression of SFAs and the delivery of SGA infants are not well established. Evidence from previous studies on animal models (mice, dams, rabbits, and swine) showed opposing results. Some investigations support early effects of unbalanced high fat (HF) diets on offspring development, causing impaired intrauterine growth and low birth-weight offspring [24,25,26]; conversely, others found foetal overgrowth [27] or no difference with the control group, in response to a maternal HF diet [28].

In general, FAs interact with the human placenta and initiate several cascade events, differing with respect to their carbon length and degree of saturation [29,30]. They can influence foetal growth through different mechanisms: (1) by altering their own specific transfer from the mother to the foetus [31]; (2) by regulating trophoblast amino acid transport through the modulation of the mammalian target of rapamycin (mTOR) and insulin-like growth factor (IGF) pathways [29,32]; and (3) by initiating innate immune responses via the toll/like receptor-4 [30]. These mechanisms has not been extensively explained in SFAs and MUFAs, for which research mainly focused on palmitic, stearic, and oleic acids [29,30].

In particular, it has been hypothesised that the high long chain saturated fat diet, known to cause insulin resistance, could be an inciting factor in the decreased expression of the embryonic IGF-1 receptor which manifests later through differences in offspring size, growth patterns, and metabolic response [24]. Alternatively, this diet may affect *Igf1r* (IGF-I receptor gene) expression in the blastocysts, resulting in subsequent insulin resistance [24]. Interestingly, Yang et al. showed how the saturated FAs palmitic acid and stearic acid play a dynamic role in the placenta inflammation status [30]. Moreover, Lager et al. found DHA and oleic acid to be associated to a decrease and increase of amino acids transfers, respectively. Palmitic acid (C16:0) was not found to affect trophoblast amino acid transport [29].

Our results on SFAs suggest a possible enhanced transfer of these fatty acids from mothers to the foetus in SGA infants, perhaps in relation their increased representation in the former, likely contributing to an altered placental metabolism as well as an impaired placental nutrient transport capacity, with consequent reduced foetal growth. Our findings mainly refer to myristic, pentadecanoic, heptadecanoic, and erucic acids, whose roles have not been investigated yet.

The present study has strengths as well as limitations. The main strengths include the measurement of erythrocyte FAs, the comparison between the three classes of infants (SGA, AGA, and LGA groups) and the large sample size. However, we were not able to measure other factors or residual confounds, such as genetic variability, which may have affected erythrocyte lipid profiles; we were also unable to rule out whether the influence of maternal fatty acid profiles on the new-borns’ body size reflects dietary differences. In our series, the use of FAs concentrations in the analyses instead of percentages did not affect results as far as FAs classes are concerned. Therefore, we used percentages in order to allow comparisons to previous studies. Nevertheless, this may be considered a limitation of the study.

## 5. Conclusions

As far as we know, the “Feeding Low-Grade Inflammation and Insulin Resistance of the Foetus” study is one of the largest cohort studies investigating the association between maternal/foetal erythrocyte fatty acid profiles and birth weight. It is worth mentioning that this was also the first study comparing maternal and foetal erythrocyte FAs in the three classes of AGA, SGA, and LGA infants.

Our study results has shown that both maternal and foetal FAs profiles may affect foetal growth during pregnancy, likely by modulating placenta metabolism. Excess of SFAs, trans, and erucic acid may play a role to shape the risk of low intrauterine growth. 

## Figures and Tables

**Table 1:** Maternal and infant characteristics.

	Variable	*n* = 607
**Mothers**	Age (years)	33.0 (29.0–37.0) ^1^
	Pre–pregnancy Body Mass Index (kg/m^2^)	22.0 (20.0–24.6) ^1^
	Underweight	41 (6.8) ^2^
	Normal weight	427 (70.3) ^2^
	Overweight	93 (15.3) ^2^
	Obese	46 (7.6) ^2^
	Gestational Weight Gain (GWG)	13.0 (10.0–16.0) ^1^
	Inadequate GWG	166 (27.4) ^2^
	Adequate GWG	243 (40.0) ^2^
	Excessive GWG	198 (32.6) ^2^
	Maternal smoking in pregnancy	93 (15.3) ^2^
	Parity	
	0	327 (53.9) ^2^
	1	224 (36.9) ^2^
	≥2	56 (9.2) ^2^
	Gestational age (weeks)	39.0 (38.0–40.0) ^1^
	Delivery method	
	Vaginal	352 (58.0) ^2^
	Caesarean	255 (42.0) ^2^
	Education level	
	Primary school	6 (0.2) ^2^
	Secondary school	82 (13.6) ^2^
	High school	284 (47.3) ^2^
	Bachelor degree	228 (37.9) ^2^
**Infants**	Infant sex: male	316 (52.1) ^2^
	Birth Weight (BW) (g)	3310 (3020–3610) ^1^
	Birth crown-heel length (BL) (cm)	50.0 (49.0–52.0) ^1^
	Head Circumference (cm)	35.0 (34.0–36.0) ^1^
	BW SDS	0.1 (−0.6–0.7) ^1^
	BL SDS	0.2 (−0.4–1.0) ^1^
	Small for Gestational Age	49 (8.2) ^2^
	Adequate for Gestational Age	483 (80.5) ^2^
	Large for Gestational Age	68 (11.3) ^2^

Data are expressed as median and interquartile range (IQR) ^1^ or as number and percentage (%) ^2^; BL, birth crown–heel length; BW, birth weight; GWG, gestational weight gain; HC, head circumference; LGA, large for gestational age; SDS, standard deviation score; SGA, small for gestational age. Anthropometric measures were taken at study enrolment for mothers, and at birth for infants.

**Table 2:** Adjusted association between maternal/infant lipid profile and infant birth weight.

Dependent Variable	Independent Variable	Coefficient	95% CI		*p*
			Lower Bound	Upper Bound	
Birth weight	Foetal C14:0	−65.41	−106.43	−14.38	0.002
	Pre-pregnancy BMI	11.19	3.47	18.91	0.005
	Excessive GWG	143.85	59.38	228.31	0.001
	Gestational age	118.60	91.29	145.92	0.000
	Infant sex (Reference: male)	−203.50	−272.56	−134.44	0.000
	Foetal C17:0	−96.5	−176.79	−16.22	0.019
	Pre-pregnancy BMI	8.44	0.74	16.14	0.032
	Excessive GWG	123.60	40.10	207.09	0.004
	Infant sex (Reference: male)	−189.37	−257.68	−121.06	0.000
	Parity (≥2)	168.24	44.22	292.26	0.008
	Maternal C18:2, n-6 (LA)	18.66	4.51	32.81	0.010
	Pre-pregnancy BMI	9.74	1.95	17.53	0.014
	Excessive GWG	138.27	53.82	222.71	0.001
	Gestational age	132.03	103.94	160.12	0.000
	Infant sex (Reference: male)	−216.29	−285.36	−147.23	0.000
	Parity (≥2)	146.58	21.20	271.96	0.022
	Maternal C20:4, n-6 (AA)	12.65	4.09	21.21	0.004
	Pre-pregnancy BMI	8.61	1.09	16.12	0.025
	Excessive GWG	131.49	50.36	212.63	0.002
	Gestational age	1651.53	204.34	3098.73	0.025
	Gestational age square	−19.42	−37.88	−0.95	0.039
	Infant sex (Reference: male)	−189.22	−255.73	−122.71	0.000
	Parity (≥2)	144.95	23.79	266.11	0.019
	Foetal C22:6, n-3 (DHA)	−22.82	−44.27	−1.37	0.037
	Maternal C22:6, n-3 (DHA)	29.73	8.10	51.36	0.007
	Excessive GWG	160.71	80.86	240.55	0.000
	Gestational age	127.85	100.43	155.28	0.000
	Infant sex (Reference: male)	−199.46	−266.89	−132.03	0.000
	Parity (≥2)	174.14	52.63	295.64	0.005
	Maternal Total PUFAs	8.42	3.33	13.51	0.001
	Pre-pregnancy BMI	8.97	1.55	16.39	0.018
	Excessive GWG	145.97	65.71	226.22	0.000
	Gestational age	1557.03	129.98	2984.075	0.033
	Gestational age square	−18.28	−36.49	−0.07	0.049
	Infant sex (Reference: male)	−187.05	−252.79	−121.31	0.000
	Parity (≥2)	164.68	45.10	284.27	0.007

Multivariable quantile regressions between infant birth weight (dependent variable) and maternal and foetal fatty acids (FAs) (independent co-variables). Analyses were adjusted for: gestational weight gain (GWG), pre-pregnancy body mass index (BMI), smoking, maternal age, offspring sex, gestational age, and parity. A separate quantile regression analysis was conducted for each FA and the final multivariable models were determined through a backward approach. Continuous variables: pre-pregnancy BMI, maternal age, gestational age. Categorical variables: GWG (Reference: adequate), smoking (Reference: no), Offspring sex (Reference: male), Parity (Reference: 0). AA, arachidonic acid; BMI, body mass index; DHA, docosahexaenoic acid; FAs: fatty acids; GWG, gestational weight gain; LA, linoleic acid; PUFAs, polyunsaturated fatty acids; Total PUFAs include: C18:2, n-6; C18:3, n-6; C18:3, n-3; C20:2, n-6; C20:3, n-6; C20:4, n-6; C20:3, n-3; C20:5, n-3; trans C22:2, n-7; C22:3, n-3; C22:4, n-5; C22:5, n-3. C22:6, n-3.

**Table 3:** Maternal lipid profile in Small for Gestational Age, Adequate for Gestational Age, and Large for Gestational Age infants.

		SGA (*n* = 49)				AGA (*n* = 483)				LGA (*n* = 68)				
		Mean	Median	25° Percentile	75° Percentile	Mean	Median	25° Percentile	75° Percentile	Mean	Median	25° Percentile	75° Percentile	*p*
**Maternal FAs**	**C12:0**	1.85	1.83	0.00	2.74	1.21	0.84	0.00	2.17	1.25	1.28	0.00	2.20	0.123 *
	**C13:0**	0.58	0.00	0.00	0.00	0.53	0.00	0.00	0.72	0.55	0.00	0.00	0.73	0.887
	**C14:0**	1.68	1.51	1.05	2.01	1.04	0.91	0.52	1.46	1.13	1.02	0.64	1.48	0.000 *^,^ᶧ
	**C15:0**	0.92	0.82	0.37	1.05	0.60	0.49	0.26	0.82	0.65	0.58	0.30	0.85	0.017 *
	**C16:0**	20.12	19.04	17.29	22.67	19.82	19.69	16.81	22.79	19.82	20.15	17.89	21.58	0.970
	**C17:0**	0.58	0.54	0.23	0.68	0.53	0.47	0.24	0.67	0.53	0.50	0.23	0.72	0.568
	**C18:0**	13.41	13.00	11.32	14.80	13.56	13.27	11.36	15.52	13.64	13.46	11.85	14.91	0.832
	**C19:0**	0.04	0.00	0.00	0.00	0.06	0.00	0.00	0.00	0.03	0.00	0.00	0.00	0.706
	**C:20**	0.32	0.39	0.00	0.48	0.32	0.39	0.00	0.48	0.38	0.41	0.26	0.53	0.322
	**C:22**	1.28	1.46	1.20	1.66	1.13	1.37	0.78	1.61	1.22	1.40	1.14	1.57	0.235
	**C24:0**	3.93	4.27	3.81	4.81	3.43	4.04	2.91	4.66	3.84	4.22	3.75	4.76	0.063
	**C12:1, n-1**	1.46	1.37	0.00	2.27	0.94	0.47	0.00	1.66	1.01	0.90	0.00	1.68	0.069 *
	**C14:1, n-5**	1.33	1.13	0.00	1.92	0.85	0.62	0.00	1.37	1.10	0.59	0.00	1.78	0.033 *
	**C15:1,** **n-** **1**	0.15	0.00	0.00	0.24	0.40	0.00	0.00	0.34	0.16	0.00	0.00	0.30	0.355
	**trans C16:1,** **n-** **7**	0.34	0.00	0.00	0.33	0.25	0.00	0.00	0.31	0.26	0.00	0.00	0.35	0.916
	**C16:1,** **n-** **7**	0.48	0.38	0.00	0.73	0.69	0.47	0.00	0.88	0.60	0.52	0.00	0.87	0.614
	**C17:1,** **n-** **7**	0.42	0.34	0.00	0.77	0.64	0.00	0.00	0.88	0.45	0.00	0.00	0.78	0.762
	**trans C18:1,** **n-** **9**	0.42	0.00	0.00	0.21	0.31	0.00	0.00	0.00	0.30	0.00	0.00	0.00	0.469
	**transC18:1,** **n-** **7**	1.61	0.00	0.00	0.00	2.87	0.00	0.00	1.97	3.69	0.00	0.00	8.55	0.283
	**C18:1,** **n-** **9**	8.47	9.49	6.71	11.41	7.88	9.60	0.89	12.54	7.32	9.58	0.97	11.52	0.749
	**C18:1,** **n-** **7**	0.95	0.98	0.74	1.25	1.29	1.01	0.70	1.26	1.27	1.00	0.82	1.22	0.952
	**C19:1, n-9**	0.07	0.00	0.00	0.00	0.20	0.00	0.00	0.25	0.12	0.00	0.00	0.19	0.040 *
	**C20:1, n-15**	0.10	0.00	0.00	0.00	0.06	0.00	0.00	0.00	0.03	0.00	0.00	0.00	0.372 ᶧ
	**C20:1, n-12**	0.09	0.00	0.00	0.21	0.17	0.00	0.00	0.35	0.18	0.04	0.00	0.34	0.104 *^,^ᶧ
	**C20:1, n-9**	0.18	0.12	0.00	0.31	0.15	0.00	0.00	0.30	0.17	0.13	0.00	0.30	0.401
	**C22:1, n-9**	0.13	0.13	0.00	0.22	0.08	0.00	0.00	0.15	0.10	0.00	0.00	0.13	0.026 *^,^ᶧ
	**C24:1, n-9**	4.61	5.80	3.30	6.26	4.34	5.18	1.50	6.21	4.29	5.22	1.48	5.93	0.604
	**C18:2, n-6 (LA)**	3.89	4.93	2.28	5.57	4.54	5.28	2.91	6.23	4.78	5.45	2.79	6.40	0.037 *^,^ᶧ
	**C18:3, n-6**	0.49	0.21	0.00	0.33	0.47	0.15	0.00	0.27	0.25	0.17	0.00	0.27	0.305
	**C18:3, n-3 (ALA)**	0.10	0.00	0.00	0.14	0.07	0.00	0.00	0.13	0.06	0.00	0.00	0.11	0.211
	**C20:2, n-6**	0.21	0.18	0.00	0.28	0.21	0.16	0.00	0.33	0.23	0.20	0.00	0.37	0.564
	**C20:3, n-6 (DGLA)**	0.82	0.99	0.28	0.19	0.94	1.08	0.25	1.33	1.07	1.13	0.43	1.62	0.135
	**C20:4, n-6 (AA)**	4.67	5.74	0.93	7.20	5.62	6.58	1.11	8.76	6.16	7.28	1.52	9.35	0.057 ᶧ
	**C20:3, n-3**	0.58	0.00	0.00	0.00	0.84	0.00	0.00	0.11	1.01	0.00	0.00	0.08	0.565
	**C20:5, n-3 (EPA)**	0.14	0.00	0.00	0.12	0.28	0.20	0.00	0.34	0.22	0.16	0.00	0.32	0.003 *^,^ᶧ
	**trans C22:2, *n-7***	0.40	0.37	0.00	0.59	0.47	0.43	0.20	0.63	0.56	0.53	0.36	0.80	0.023 ᶧ^,^ᶱ
	**C22:5, *n-3* (DPA)**	1.54	0.81	0.00	2.13	0.92	.51	0.00	1.19	0.86	0.53	0.00	1.03	0.108
	**C22:6, *n-3* (DHA)**	3.22	2.93	1.42	4.55	3.34	3.00	1.97	4.59	3.20	3.13	1.85	4.47	0.868
	**Total FAs**	100.00	100.00	100.00	100.00	99.79	100.00	100.00	100.00	100.00	100.00	100.00	100.00	0.999
	**Total SFAs**	44.71	43.93	39.65	48.44	42.25	42.01	37.65	46.88	43.03	42.82	40.00	46.45	0.061 *
	**Total MUFAs**	21.69	22.00	19.18	23.72	21.76	21.52	18.56	24.53	21.24	21.41	18.49	23.83	0.739
	**Total PUFAs**	17.20	18.43	12.48	22.18	18.95	19.61	14.75	23.48	19.80	21.41	16.09	24.91	0.106
	**Total *n-3***	5.57	4.65	2.25	8.52	5.44	4.78	3.02	7.01	5.35	4.61	2.71	5.86	0.743
	***Total n-6***	9.87	11.80	6.09	13.80	11.56	12.96	7.44	15.50	12.25	13.70	8.62	16.14	0.026 *^,^ᶧ

Percentage of FAs in maternal and foetal erythrocytes of SGA, AGA and LGA infants. AA, arachidonic acid; ALA, alpha-linolenic acid; DGLA, dihomo-gamma-linolenic acid; DHA, docosahexaenoic acid; DPA, docosapentaenoic acid; EPA, eicosapentaenoic acid; LA, linoleic acid; FAs, fatty acids; MUFAs, monounsaturated fatty acids; Perc., percentiles; PUFAs, polyunsaturated fatty acids; SFAs, saturated fatty acids. Total SFAs include: C12:0, C13:0, C14:0, C15:0, C16:0, C17:0, C18:0, C19:0, C20:0, C22:0, C24:0; Total MUFAs include: C12:1 n-1, C14:1 n-5, C15:1 n-1, trans C16:1 n-7, C16:1 n-7, C17:1 n-7, trans C18-1 n-9, C18:1 n-9, trans C18-1 n-7, C18:1 n-7, C19:1 n-9, C20:1 n-15, C20:1 n-12, C20:1 n-9, C22:1 n-9, C24:1 n-9. Total PUFAs include: C18:2 n-6, C18:3 n-6, C18:3 n-3, C20:2 n-6, C20:3 n-6, C20:4 n-6, C20:3 n-3, C20:5 n-3, trans C22:2 n-7, C22:5 n-3, C22:6 n-3. Total n-3 include: C18:3 n-3, C20:3 n-3, C20:5 n-3, C22:5 n-3, C22:6 n-3. Total n-6 include: C18:2 n-6, C18:3 n-6, C20:3 n-6, C20:4 n-6. Kruskal–Wallis test was used to compare FAs in the SGA, AGA, and LGA groups. * Significant difference between SGA and AGA; ᶧ significant difference between SGA and LGA; ᶱ significant difference between AGA and LGA.

**Table 4:** Foetal lipid profile in Small for Gestational Age, Adequate for Gestational Age, and Large for Gestational Age infants.

		SGA (*n* = 49)				AGA (*n* = 483)				LGA (*n* = 68)				
		Mean	Median	25° Percentile	75° Percentile	Mean	Median	25° Percentile	75° Percentile	Mean	Median	25° Percentile	75° Percentile	*p*
**Foetal FAs**	**C12:0**	1.76	1.63	0.00	2.73	1.27	0.81	0.00	2.26	1.31	1.13	0.00	2.32	0.136
	**C13:0**	0.79	0.00	0.00	1.54	0.53	0.00	0.00	0.84	0.81	0.00	0.00	1.07	0.585
	**C14:0**	1.51	1.39	0.62	1.90	1.02	0.90	0.50	1.43	1.15	1.01	0.72	1.43	0.009 *
	**C15:0**	0.85	0.72	0.33	1.09	0.57	0.45	0.23	0.77	0.59	0.34	0.13	0.67	0.002 *^,^ᶧ
	**C16:0**	19.32	19.27	17.08	21.93	19.20	19.34	16.33	22.18	18.96	19.08	16.33	21.47	0.833
	**C17:0**	0.65	0.55	0.39	0.74	0.48	0.43	0.23	0.58	0.44	0.38	0.21	0.58	0.003 *^,^ᶧ
	**C18:0**	13.43	13.99	11.53	15.30	14.11	14.10	11.92	16.14	14.02	13.92	12.13	15.63	0.576
	**C19:0**	0.03	0.00	0.00	0.00	0.04	0.00	0.00	0.00	0.01	0.00	0.00	0.00	0.777
	**C20:0**	0.35	0.45	0.00	0.55	0.36	0.44	0.00	0.57	0.37	0.45	0.00	0.54	0.972
	**C22:0**	1.10	1.20	1.04	1.44	0.96	1.17	0.59	1.35	0.98	1.19	0.76	1.34	0.230
	**C24:0**	3.56	4.01	3.19	4.69	3.41	3.98	2.30	4.70	3.65	4.03	3.12	4.62	0.805
	**C12:1, n-1**	1.31	0.71	0.00	2.18	0.97	0.47	0.00	1.71	0.98	0.69	0.00	1.73	0.484
	**C14:1, n-5**	1.29	1.16	0.00	1.69	0.79	0.56	0.00	1.31	1.11	0.72	0.00	1.65	0.018 *
	**C15:1,** **n-** **1**	0.22	0.00	0.00	0.14	0.20	0.00	0.00	0.26	0.20	0.00	0.00	0.16	0.272
	**trans C16:1, n-7**	0.38	0.08	0.00	0.60	0.31	0.00	0.00	0.50	0.19	0.00	0.00	0.31	0.113 ᶧ
	**C16:1,** **n-** **7**	0.32	0.00	0.00	0.72	0.48	0.40	0.00	0.73	0.73	0.46	0.00	0.76	0.196
	**C17:1,** **n-** **7**	0.35	0.25	0.00	0.66	0.53	0.00	0.00	0.64	0.29	0.00	0.00	0.61	0.322
	**trans C18:1,** **n-** **9**	0.63	0.00	0.00	0.23	0.26	0.00	0.00	0.00	0.26	0.00	0.00	0.05	0.272 *
	**trans C18:1,** **n-** **7**	1.87	0.00	0.00	0.25	2.35	0.00	0.00	6.08	2.37	0.00	0.00	6.10	0.718
	**C18:1,** **n-** **9**	5.69	6.50	1.56	8.49	5.46	6.68	0.00	8.85	5.39	6.62	0.55	8.55	0.948
	**C18:1,** **n-** **7**	1.69	1.55	1.09	1.84	1.54	1.37	1.02	1.71	1.63	1.43	1.11	1.68	0.307
	**C19:1, n-9**	0.10	0.00	0.00	0.00	0.17	0.00	0.00	0.19	0.13	0.00	0.00	0.19	0.213
	**C20:1, n-15**	0.13	0.00	0.00	0.00	0.05	0.00	0.00	0.00	0.09	0.00	0.00	0.00	0.773
	**C20:1, n-12**	0.15	0.00	0.00	0.12	0.12	0.06	0.00	0.18	0.14	0.14	0.00	0.20	0.025 ᶧ^,^ᶱ
	**C20:1, n-9**	0.12	0.00	0.00	0.23	0.10	0.00	0.00	0.17	0.12	0.00	0.00	0.17	0.807
	**C22:1, n-9**	0.28	0.00	0.00	0.12	0.14	0.00	0.00	0.15	0.09	0.00	0.00	0.15	0.854
	**C24:1, n-9**	3.25	3.80	1.64	4.73	3.09	3.69	0.72	4.44	3.02	3.45	0.62	4.37	0.705
	**C18:2, n-6 (LA)**	2.07	2.11	1.65	2.57	2.27	2.25	1.75	2.75	2.07	2.11	1.61	2.54	0.203
	**C18:3, n-6**	0.38	0.14	0.00	0.39	0.27	0.11	0.00	0.24	0.22	0.12	0.00	0.22	0.273
	**C18:3, n-3 (ALA)**	0.06	0.00	0.00	0.00	0.07	0.00	0.00	0.00	0.04	0.00	0.00	0.00	0.616
	**C20:2, n-6**	0.30	0.35	0.00	0.48	0.46	0.50	0.00	0.67	0.57	0.59	0.35	0.78	0.001 *^,^ᶱ^,^ᶧ
	**C20:3, n-6 (DGLA)**	1.18	1.27	0.88	1.74	1.27	1.44	0.56	1.83	1.31	1.51	0.34	1.81	0.449
	**C20:4, n-6 (AA)**	6.28	7.22	2.55	8.82	6.59	7.79	1.90	9.71	6.90	8.24	1.22	10.09	0.527
	**C20:3, n-3**	0.64	0.00	0.00	0.07	1.05	0.00	0.00	0.10	1.14	0.00	0.00	0.08	0.836
	**C20:5, n-3 (EPA)**	0.17	0.00	0.00	0.18	0.21	0.12	0.00	0.25	0.18	0.15	0.00	0.29	0.072 *^,^ᶧ
	**trans C22:2, n-7**	0.24	0.14	0.00	0.39	0.29	0.21	0.00	0.46	0.29	0.30	0.00	0.48	0.614
	**C22:5, n-3 (DPA)**	1.15	0.50	0.00	1.37	0.65	0.34	0.00	0.67	0.69	0.22	0.00	0.61	0.058 *^,^ᶧ
	**C22:6, n-3 (DHA)**	3.90	3.42	2.19	5.18	3.45	3.19	2.18	4.38	3.19	3.06	2.18	3.94	0.573
	**Total FAs**	100.00	100.00	100.00	100.00	100.00	100.00	100.00	100.00	100.00	100.00	100.00	100.00	1000
	**Total SFAs**	43.43	43.10	39.57	47.93	42.01	42.19	37.55	47.04	42.29	42.01	38.34	46.17	0.501
	**Total MUFAs**	18.28	17.22	15.27	18.91	17.20	16.78	14.60	19.07	16.89	16.16	14.57	19.05	0.320
	**Total PUFAs**	17.82	18.06	14.35	21.73	18.04	18.45	14.54	22.08	18.18	18.82	14.98	21.82	0.890
	**Total *n*-3**	5.85	4.92	2.87	8.05	5.39	4.44	3.10	6.86	5.25	4.32	3.11	5.87	0.732
	**Total *n*-6**	9.92	10.82	4.99	12.89	10.40	11.92	5.01	14.21	10.50	11.70	6.29	14.23	0.584

Percentage of FAs in foetal erythrocytes of SGA, AGA, and LGA infants. AA, arachidonic acid; ALA, alpha-linolenic acid; DGLA, dihomo-gamma-linolenic acid; DHA, docosahexaenoic acid; DPA, docosapentaenoic acid; EPA, eicosapentaenoic acid; LA, linoleic acid; FAs, fatty acid; MUFAs, monounsaturated fatty acids; Perc., percentiles; PUFAs, polyunsaturated fatty acids; SFAs, saturated fatty acids. Total SFAs include: C12:0, C13:0, C14:0, C15:0, C16:0, C17:0, C18:0, C19:0, C20:0, C22:0, C24:0. Total MUFAs include: C12:1 n-1, C14:1 n-5, C15:1 n-1, trans C16:1 n-7, C16:1 n-7, C17:1 n-7, trans C18-1 n-9, C18:1 n-9, trans C18-1 n-7, C18:1 n-7, C19:1 n-9, C20:1 n-15, C20:1 n-12, C20:1 n-9, C22:1 n-9, C24:1 n-9. Total PUFAs include: C18:2 n-6, C18:3 n-6, C18:3 n-3, C20:2 n-6, C20:3 n-6, C20:4 n-6, C20:3 n-3, C20:5 n-3, trans C22:2 n-7, C22:5 n-3, C22:6 n-3. Total n-3 include: C18:3 n-3, C20:3 n-3, C20:5 n-3, C22:5 n-3, C22:6 n-3. Total n-6 include: C18:2 n-6, C18:3 n-6, C20:3 n-6, C20:4 n-6. Kruskal–Wallis test was used to compare FAs in the SGA, AGA, and LGA groups. * Significant difference between SGA and AGA; ᶧ significant difference between SGA and LGA; ᶱ significant difference between AGA and LGA.

## Supplementary Materials

The following are available online at http://www.mdpi.com/2072-6643/10/4/402/s1, Table S1: Fatty acid distribution.

## Abbreviations

The following abbreviations are used in this manuscript:

AA	Arachidonic acid
ALA	α-linolenic acid
BL	Birth crown-heel length
BMI	Body mass index
BW	Birth weight
DGLA	Dihomogamma linoleic acid
DHA	Docosaexahenoic acid
DPA	Docosapentaenoic acid
EPA	Ecosapentaenoic acid
FAs	Fatty acids
GWG	Gestational weight gain
HC	Head circumference
HF	High fat
IGFs	Insulin-like growth factors
LA	Linoleic acid
LC-PUFAs	Long chain unsaturated fatty acids
mTOR	Mammalian Target of Rapamycin
MUFAs	Monounsaturated fatty acids
OPBG	*Ospedale Pediatrico Bambino Gesù*
PUFAs	Polyunsaturated fatty acids
SCH	*San Camillo Forlanini* Hospital
SD	Standard Deviation
SDS	Standard deviation score
SES	Socio-economic status
SFAs	Saturated fatty acids
WHO	World Health Organization
