# Supplementary material for: Association between Maternal and Foetal Erythrocyte Fatty Acid Profiles and Birth Weight
Source: Nutrients. 2018 Mar 23;10(4):402. doi: 10.3390/nu10040402 (PMC5946187; doi:10.3390/nu10040402)
Supplement: Supplementary file 1 [file nutrients-10-00402-s001.pdf]

Tabel S1. Maternal and foetal lipid profile distribution

	Maternal					Foetal				
	**Mean**	**Median**	**25° Percentile**	**75° Percentile**	**p***	**Mean**	**Median**	**25° Percentile**	**75° Percentile**	**p***
**C12:0**	1.28	0.91	0.00	2.27	**0.000**	1.32	0.89	0.00	2.31	**0.000**
**C13:0**	0.54	0.00	0.00	0.72	**0.000**	0.59	0.00	0.00	0.88	**0.000**
**C14:0**	1.10	0.96	0.56	1.51	**0.000**	1.08	0.93	0.54	1.46	**0.000**
**C15:0**	0.64	0.52	0.27	0.85	**0.000**	0.60	0.45	0.22	0.79	**0.000**
**C16:0**	19.79	19.67	16.99	22.50	**0.000**	19.16	19.25	16.32	22.02	**0.000**
**C17:0**	0.53	0.47	0.24	0.67	**0.000**	0.49	0.43	0.23	0.60	**0.000**
**C18:0**	13.53	13.27	11.41	15.42	**0.000**	14.03	14.03	11.87	16.08	**0.000**
**C19:0**	0.05	0.00	0.00	0.00	**0.000**	0.04	0.00	0.00	0.00	**0.000**
**C:20**	0.33	0.39	0.00	0.48	**0.014**	0.36	0.44	0.00	0.56	**0.000**
**C:22**	1.15	1.38	0.96	1.61	**0.000**	0.97	1.17	0.75	1.36	**0.000**
**C24:0**	3.53	4.10	3.12	4.68	**0.000**	3.46	4.00	2.74	4.70	**0.000**
**C12:1, n-1**	0.99	0.59	0.00	1.74	**0.000**	1.00	0.56	0.00	1.78	**0.000**
**C14:1, n-5**	0.91	0.65	0.00	1.45	**0.000**	0.86	0.66	0.00	1.38	**0.000**
**C15:1, n-1**	0.35	0.00	0.00	0.31	**0.000**	0.20	0.00	0.00	0.26	**0.000**
***trans* C16:1, n-7**	0.26	0.00	0.00	0.31	**0.000**	0.30	0.00	0.00	0.50	**0.000**
**C16:1, n-7**	0.66	0.46	0.00	0.86	**0.000**	0.49	0.39	0.00	0.73	**0.000**
**C17:1, n-7**	0.60	0.00	0.00	0.85	**0.000**	0.49	0.00	0.00	0.63	**0.000**
***trans* C18:1, n-9**	0.32	0.00	0.00	0.00	**0.000**	0.29	0.00	0.00	0.00	**0.000**
***trans* C18:1, n-7**	2.88	0.00	0.00	5.22	**0.000**	2.31	0.00	0.00	5.77	**0.000**
**C18:1, n-9**	7.83	9.52	1.01	12.45	**0.000**	5.45	6.63	0.00	8.73	**0.000**
**C18:1, n-7**	1.25	1.00	0.71	1.25	**0.000**	1.56	1.39	1.04	1.71	**0.000**
**C19:1, n-9**	0.18	0.00	0.00	0.23	**0.000**	0.15	0.00	0.00	0.18	**0.000**
**C20:1, n-15**	0.06	0.00	0.00	0.00	**0.000**	0.06	0.00	0.00	0.00	**0.000**
**C20:1, n-12**	0.16	0.00	0.00	0.34	**0.000**	0.12	0.06	0.00	0.18	**0.000**

**C20:1, n-9**	0.16	0.00	0.00	0.31	**0.000**	0.10	0.00	0.00	0.17	**0.000**
**C22:1, n-9**	0.09	0.00	0.00	0.17	**0.000**	0.14	0.00	0.00	0.15	**0.000**
**C24:1, n-9**	4.38	5.22	1.52	6.21	**0.000**	3.10	3.69	0.72	4.44	**0.000**
**C18:2, n-6 (LA)**	4.52	5.27	2.80	6.18	**0.000**	2.23	2.23	1.72	2.71	**0.000**
**C18:3, n-6**	0.45	0.15	0.00	0.29	**0.000**	0.28	0.12	0.00	0.25	**0.000**
**C18:3, n-3 (ALA)**	0.07	0.00	0.00	0.13	**0.000**	0.06	0.00	0.00	0.00	**0.000**
**C20:2, n-6**	0.21	0.18	0.00	0.33	**0.000**	0.46	0.49	0.00	0.67	**0.000**
**C20:3, n-6 (DGLA)**	0.95	1.08	0.27	1.35	**0.000**	1.28	1.45	0.62	1.82	**0.000**
**C20:4, n-6 (AA)**	5.63	6.63	1.17	8.75	**0.000**	6.63	7.80	1.93	9.76	**0.000**
**C20:3, n-3**	0.83	0.00	0.00	0.09	**0.000**	1.01	0.00	0.00	0.10	**0.000**
**C20:5, n-3 (EPA)**	0.26	0.18	0.00	0.33	**0.000**	0.20	0.12	0.00	0.25	**0.000**
**trans C22:2, n-7**	0.48	0.44	0.23	0.64	**0.000**	0.29	0.20	0.00	0.46	**0.000**
**C22:5, n-3 (DPA)**	0.98	0.59	0.00	1.24	**0.000**	0.70	0.34	0.00	0.70	**0.000**
**C22:6, n-3 (DHA)**	3.34	3.08	1.96	4.55	**0.000**	3.48	3.20	2.19	4.35	**0.000**
**Total FAs**	99.83	100.00	100.00	100.00	**0.000**	100.00	100.00	100.00	100.00	0.852
**Total SFAs**	42.48	42.14	38.10	46.74	**0.000**	42.13	42.19	37.80	47.03	**0.000**
**Total MUFAs**	21.67	21.52	18.56	24.30	**0.000**	17.22	16.61	14.60	19.03	**0.000**
**Total PUFAs**	18.99	19.83	14.83	23.59	**0.000**	18.10	18.51	14.54	22.12	**0.003**
**Total n-3**	5.48	4.79	3.00	7.04	**0.000**	5.43	4.45	3.10	7.10	**0.000**
**Total n-6**	11.53	12.96	7.50	15.48	**0.000**	10.42	11.81	5.29	14.21	**0.000**

AA, arachidonic acid; ALA, alpha-linolenic acid; DGLA, dihomo-gamma-linolenic acid; DHA, docosahexaenoic acid; DPA, docosapentaenoic acid; EPA, eicosapentaenoic acid; LA, linoleic acid; FAs, fatty acid methyl ester; MUFAs, monounsaturated fatty acids; Perc., percentiles; PUFAs, polyunsaturated fatty acids; SFAs, saturated fatty acids; Total SFAs include: C12:0, C13:0, C14:0, C15:0, C16:0, C17:0, C18:0, C19:0, C20:0, C22:0, C24:0; Total MUFAs include: C12:1 n-1, C14:1 n-5, C15:1 n-1, trans C16:1 n-7, C16:1 n-7, C17:1 n-7, trans C18-1 n-9, C18:1 n-9, trans C18-1 n-7, C18:1 n-7, C19:1 n-9, C20:1 n-15, C20:1 n-12, C20:1 n-9, C22:1 n-9, C24:1 n-9; Total PUFAs include: C18:2 n-6, C18:3 n-6, C18:3 n-3, C20:2 n-6, C20:3 n-6, C20:4 n-6, C20:3 n-3, C20:5 n-3, trans C22:2 n-7, C22:5 n-3, C22:6 n-3; Total n-3 include: C18:3 n-3, C20:3 n-3, C20:5 n-3, C22:5 n-3, C22:6 n-3; Total n-6 include: C18:2 n-6, C18:3 n-6, C20:3 n-6, C20:4 n-6. * Skewness – Kurtosis test to evaluate FAs distribution. Variable are considered non normally distributed for $p<0.05$.
